# Changes of phenolic secondary metabolite profiles in the reaction of narrow leaf lupin (*Lupinus angustifolius*) plants to infections with *Colletotrichum lupini* fungus or treatment with its toxin

**DOI:** 10.1007/s11306-012-0475-8

**Published:** 2012-10-30

**Authors:** Anna Wojakowska, Dorota Muth, Dorota Narożna, Cezary Mądrzak, Maciej Stobiecki, Piotr Kachlicki

**Affiliations:** 1 Institute of Bioorganic Chemistry PAS, Noskowskiego 12/14, 61-704 Poznań, Poland; 2 Institute of Plant Genetics PAS, Strzeszyńska 34, 60-479 Poznań, Poland; 3 Department of Biochemistry and Biotechnology, Faculty of Agronomy, Poznań University of Life Science, ul. Wojska Polskiego 28, 60-637 Poznan, Poland

**Keywords:** *Colletotrichum lupini*, Elicitor, Flavonoids, Infection, Liquid chromatography/Mass spectrometry, *Lupinus angustifolius*, Secondary metabolite profiling

## Abstract

Plant interactions with environmental factors cause changes in the metabolism and regulation of biochemical and physiological processes. Plant defense against pathogenic microorganisms depends on an innate immunity system that is activated as a result of infection. There are two mechanisms of triggering this system: basal immunity activated as a result of a perception of microbe-associated molecular patterns through pattern recognition receptors situated on the cell surface and effector-triggered immunity (ETI). An induced biosynthesis of bioactive secondary metabolites, in particular phytoalexins, is one of the mechanisms of plant defense to fungal infection. Results of the study on narrow leaf lupin (*Lupinus angustifolius* L.) plants infected with the anthracnose fungus *Colletotrichum lupini* and treated with fungal phytotoxic metabolites are described in the paper. The *C. lupini* phytotoxins were isolated from liquid cultures, purified and partially characterized with physicochemical methods. Accumulation of secondary metabolites on leaf surface and within the tissues of plants either infected, treated with the fungal phytotoxin or submitted to both treatments was studied using GC-MS and LC-MS, respectively. Substantial differences in isoflavone aglycones and glycoconjugate profiles occurred in response to different ways of plant treatment.

**Electronic supplementary material:**

The online version of this article (doi:10.1007/s11306-012-0475-8) contains supplementary material, which is available to authorized users.

## Introduction

Flavonoids and their conjugates play an important role in Fabaceae family plants (Gould and Lister 2006). Among other functions, they are defense secondary metabolites synthesized as the result of fungal infection or stresses of other kinds (Bednarek et al. 2003; Lozovaya et al. 2004; Farag et al. 2008, Schliemann et al. 2008; Jasiński et al. 2009). Many classes of these polyphenolic metabolites have an antibiotic activity during plant interactions with pathogenic microorganisms (Dixon and Paiva 1995; Treutter 2006), and these compounds play the roles of phytoalexins or phytoanticipins in plant tissues (van Etten et al. 1994).

Plant defense against pathogenic microorganisms depends on an innate immunity system that is activated as the result of infection. There are two mechanisms of triggering this system: basal immunity activated as a result of microbe-associated molecular patterns (MAMPs) being perceived through pattern recognition receptors (PRR) situated on the cell surface and effector-triggered immunity (Boller and Felix 2009). The effector-triggered immunity, sometimes called gene-for-gene resistance, often leads to hypersensitive response of plants and formation of necrotic spots around the infection site due to the programmed cell apoptosis. Effectors of microbial origin, that modulate the plant response to infection, are mainly proteins or peptides, but in some circumstances, low-molecular-weight natural products may also play this role (Wolpert et al. 2002; Allwood et al. 2008; Boller and He 2009; Bednarek and Osbourn 2009; Djamei et al. 2011). Different compounds synthesized by pathogenic microorganisms (e.g. phytotoxins) or being products of pathogen or plant cell degradation are recognized by plant cells as signals for the onset of infection or its danger. Such anticipation of infection is called the defense priming (Conrath 2011) and it has been found to be inherited in the progeny of the infected plant (Luna et al. 2012). Plant reactions on the biochemical or physiological level and activation of various metabolic pathways depend on the type and origin of these signaling natural products. Different secondary metabolites are synthesized after perception and recognition of the signals originating from plant or pathogenic microorganism elicitors created during the first steps of plant defense reactions (Boller and Felix 2009; Grotewold 2005; Veitch 2009). Biosynthesis of isoflavone and closely related pterocarpan phytoalexins by plants from the Fabaceae family is of interest in this respect. Medicarpin synthesized by plants from the *Medicago* species, pisatin in *Pisum* or glyceollins in soybean (*Glycine max*), and luteone and wighteone in *Lupinus* are among the best characterized compounds.

Problems connected with various responses of the plant to different elicitors were widely described in the case of *Medicago truncatula*, the Fabaceae model plant. The yeast extract elicitor and methyl jasmonate, the wound-signaling natural product, were used to create specific responses in the cell cultures. Quantitative and qualitative changes of isoflavones and their glycoconjugates as well as substantial differences at the transcriptional level were observed in the treated cultures (Broeckling et al. 2005; Suzuki et al. 2005; Naoumkina et al. 2007; Farag et al. 2008). It was also demonstrated that various classes of flavonoids play different roles during the symbiotic interaction of *M. truncatula* plants with the nodule-forming rhizobia (Zhang et al. 2009).

*Colletotrichum* is a genus of plant pathogenic fungi that causes anthracnose disease of various plant species. The taxonomy of this genus is still not clear and about 60 species are classified within it at present (Kirk et al. 2008); however, the *Index Fungorum* (www.indexfungorum.org) lists 711 names of its species and subspecies. *C. lupini*, specific for lupin plants (Nirenberg et al. 2002), is widespread on all continents and has been detected in Poland since 1995 (Frencel 1998). *Colletotrichum* spp. are known to produce phytotoxic secondary metabolites (García-Pajón and Collado 2003; Mancilla et al. 2009) that, in many cases, induce symptoms on plants similar to those caused by the fungi themselves.

Profiles of isoflavone glycoconjugates, free isoflavone aglycones and phytoalexins in leaves of lupin plants treated with fungal phytotoxic secondary metabolites, plants infected with *C. lupini* fungus, and plants submitted successively to both treatments are compared in this study. The *C. lupini* phytotoxic metabolites were isolated and partially purified from the fungus liquid culture medium and either sprayed on the lupin plantlets or placed on the surface of wounded leaves with a microsyringe. Profiles of the target flavonoids and their glycoconjugates present inside of plant tissues or exuded to the cuticular layer were recorded with LC-MS and GC-MS systems, respectively. Numerous natural products were recognized unambiguously after comparison of the retention times (RT) and mass spectra of the compounds with those registered for standards. On the other hand, some compounds were only tentatively identified on the basis of the mass spectra recorded during the performed HPLC-ESI/MS/MS analyses, in which MS^2^ and pseudo-MS^3^ experiments were applied for structural characterization of the natural products.

## Materials and methods

### Reagents and standards

Solvents for extraction and LC-MS analyses (methanol, acetonitrile and deionized water) were HPLC or LC-MS grade; derivatization reagents for GC-MS analyses (MSTFA—*N*-methyl-*N*-(trimethylsilyl)trifluoroacetamide, methoxylamine, pyridine) were purchased from Sigma-Aldrich (Poznań, Poland). Standards of luteolin and isoflavone aglycones were obtained from Extrasynthese (Genay, France) or were isolated from lupins and characterized with different physico-chemical methods in the Laboratory (Franski et al. 1999a, b).

### Plant material

Four cultivars of narrow leaf lupin (*L. angustifolius* L.) were used in experiments (sweet cultivars—Baron, Sonet, and bitter—Karo, Mirela). The seeds were purchased from the Plant Breeding Station Przebędowo, Poland. They were sterilized with 1.5 % potassium hypochlorite for 20 min, washed several times with sterile deionized water and sown in pots with Perlite. Plants (five seedlings per pot) were grown in a greenhouse with controlled temperature (22/18 °C day/night) and light (16/8 h photoperiod) for 2 weeks.

### *Colletotrichum lupini* cultures and growth

The fungus *C. lupini* (isolate Col2) was isolated from anthracnose-diseased white lupin (*L. albus* L.) plants in Przebędowo, Poland, in 1998 and maintained in the fungal collection of the Institute of Plant Genetics, Polish Academy of Sciences. It has been identified according to Nirenberg et al. (2002). Conidial spores of the fungus were placed in a Petri dish with potato dextrose agar (PDA) medium and incubated at 28 °C for 3 weeks. Spores were collected by flooding the plate with sterile 0.1 % (v/v) Tween 20 solution, followed by filtration to remove parts of the mycelium. Spore concentrations were determined by counting and a suspension of 2 × 10^6^ spores per ml was used for spraying the plants.

### Isolation and purification of *C. lupini* phytotoxic metabolites

*Colletotrichum lupini* was grown on the modified Fries medium placed in Roux bottles (120 bottles, 200 ml of medium in each) at room temperature for 3 weeks. The culture was filtrated and the filtrate was vacuum-evaporated to about 1.5 l. Secondary metabolites were extracted 4 times with 0.5 l portions of ethyl acetate. The combined organic phases were evaporated to dryness and the residue was re-dissolved in a small volume of 25 % methanol–water solution. This solution was applied to a reversed-phase C18 flash chromatography column 1.5 × 85 cm (Sigma-Aldrich, Poznań, Poland). The column was eluted with methanol–water solutions with methanol contents increasing stepwise from 25 to 65 %. Fractions of the eluate (25 ml) were collected and a 5 μl sample from each fraction was spotted onto needle-punctured white and narrow leaf lupin (*L. albus* cv. Wat and *L. angustifolius* cv. Sonet) leaves for the phytotoxicity tests. Fractions containing phytotoxic compounds (causing the development of necrotic spots, eluted with 55 % methanol) were submitted to HPLC-MS analysis and to the preparative HPLC in an attempt to achieve pure phytotoxins. A solution containing 1 mg/ml of the mixture of the phytotoxic compounds in 10 % methanol was prepared and used for plant elicitation.

Attempts for the structural studies of the above natural products with physicochemical methods (mass spectrometry (MS), UV spectrophotometry, ^1^H, ^13^C NMR and correlation COSY, NOESY, HMBC, HSQC spectra) only allowed partial characterization of these compounds.

### Infection of lupin seedlings with *C. lupini* and elicitation with fungal toxin

Lupin plants (*L. angustifolius*) were grown in a greenhouse in pots with perlite, five seedlings per pot for 2 weeks.

#### Experiment 1

The plants at the two-leaf stage (2 weeks after germination) were infected with spores of *C. lupini* by spraying of whole plants with the spore suspension (2 × 10^6^ spores/ml, approximately 5 ml/plant), control plants were sprayed with sterile water. Seedlings were placed in hoods in order to maintain the humidity as high as possible for 24 h to initiate the infection process and, thereafter, the humidity was maintained at 70 %. Leaves were collected 24, 48, 96, 168, and 264 h after the infection, immediately frozen in liquid nitrogen, and kept at −80 °C until the analysis. Three independent inoculation experiments have been performed and the first symptoms of anthracnose were visible 7 days after the inoculation.

#### Experiment 2

The plants at the two-leaf stage were also elicited with phytotoxic secondary metabolites of *C. lupini* using two different methods. In the first experiment (“elicitation 1”—E1), 10 μl samples of the toxin solution (1 mg/ml in 10 % methanol) were spotted on leaves of the first level of seedlings wounded with a needle. The control plants were wounded and similarly treated with 10 % methanol. For the estimation of the wounding effect, untreated leaves of lupin seedlings were sprayed with 10 % methanol and collected at the same time points. Leaves of different ages (from two levels) were collected separately in three repetitions at various time points. Each repetition consisted of material from three seedlings grown in different pots in order to minimize the influence of the biological diversity. Leaves were collected 12, 24, and 48 h after the treatment. The second method of elicitation (“elicitation 2”—E2) involved spraying of whole plants with the fungal toxin solution (100 μg/ml solution in 10 % methanol, approximately 2 ml per plant), control plants were sprayed with 10 % methanol. Leaves were collected after 12, 24, and 48 h.

#### Experiment 3

The last experiment was based on plants at the two-leaf stage sprayed with the *C. lupini* phytotoxic metabolites and, after 48 h, submitted to inoculation with the fungal spores. In this experiment, four types of samples were analyzed: samples from the control plants (sprayed with water or 10 % MeOH and not inoculated with fungal spores); plants sprayed with the phytotoxins and not infected with the spores; plants subjected to infection without previous elicitation with the phytotoxin; plants subjected to both elicitation and infection. Leaves were collected from plants 216 h after the elicitation and 168 h after the infection with spores. The plant material was frozen in liquid nitrogen directly after cutting and stored at −80 °C until the extraction.

### Extraction of phenolic secondary metabolites from plant tissues

Extractions of phenolic secondary metabolites from green parts of the lupin plants were done in two independent ways. For the LC-MS analysis, the frozen leaves (100 mg fresh weight) were homogenized in 2 ml of 80 % methanol (ball mill MM200, Retsch, Haan, Germany), and the suspension was placed in an ultrasonic bath for 30 min. Luteolin was added to the homogenates as an internal standard. The extracts were centrifuged and the supernatants were transferred to new screw-cupped tubes. The solvent was removed in a vacuum concentrator at room temperature (Savant SPD 121P, Thermo Electron Corporation, Waltham, USA). Samples were dissolved in 300 μl of 80 % methanol in water and centrifuged at 10,000 rpm for 10 min, transferred to autosampler vials and immediately subjected to LC-MS analyses.

For GC-MS analysis of compounds present on lupin leaf surface, green parts of lupin plants (five plants for each sample) were washed with 100 ml of CH_2_Cl_2_ for 20 s. The washing time was optimized to avoid damage of the cells, causing a leakage of cytosolic compounds that occurred with a prolonged action of the organic solvent. Collection of surface compounds was done at different time points after elicitation or infection. The obtained solutions were evaporated, then the sample was dissolved in 2 ml of CH_2_Cl_2_ and the volume corresponding to 2 mg of the original dry weight from the each sample was transferred to the Teflon-lined screw-capped vials and taken for further GC-MS analysis. Ribitol (20 μl of methanol solution at a concentration of 1 mg/ml) was added to each sample as an internal standard and a two-stage chemical derivatization procedure was performed. Forty μl of *O*-methylhydroxylamine hydrochloride solution in pyridine (20 mg/ml) was added to the sample and heated at 40 °C for 90 min followed by addition of 70 μl MSTFA (*N*-acetyl-*N*-(trimethylsilyl)-trifluoroacetamide) and heating at 37 °C for 30 min. The sample was centrifuged at 10,000 rpm for 10 min, transferred to autosampler glass vials and subjected to the GC-MS analyses. Two biological samples were collected from each object and two independent extracts were prepared and analyzed for each sample.

### Gas chromatography/mass spectrometry

GC-MS analyses of leaf surface compounds were performed with Agilent 6890 N gas chromatograph with a 7683 autosampler (Agilent Technologies, Stockport, UK) equipped with a DB-5 column (30 m × 0.25 mm i.d., film thickness 0.25 μm) from J&W Scientific Co. (USA) and coupled to the time-of-flight mass spectroscope (MS-ToF) analyzer from Waters. Helium was used as the carrier gas at a flow rate of 1 ml/min. The GC oven temperature program was as follows: 2 min at 70 °C, raised by 10 °C/min to 300 °C, and held for 15 min at 300 °C. The total analysis time was 45 min. The injector temperature was 250 °C and 50 % of the recovered vapor was passed into the chromatography column (split 50). The interface temperature was 230 °C and source temperature was 250 °C. In-source fragmentation was performed with 70 eV energy. Mass spectra were recorded in the 50–650 *m/z* range and data were analyzed using the Waters MassLynx ver. 4.1.

### Liquid chromatography/mass spectrometry

Laboratory procedures for the extraction and analysis of secondary metabolites have been well established in our laboratory (Muth et al. 2008, 2009; Jasiński et al. 2009) and are described in details in the Supporting Information.

### PCR analysis

Total RNA was isolated from lupin leaves using SV Total RNA Isolation System (Promega) according to the manufacturer recommendations. The RNA concentration in each sample was measured using the Nanodrop 2000 spectrophotometer at 260 nm. Reverse transcription was performed with 2 μg of the total RNA used as the template with Verte M-MLV reverse transcriptase (Novazym) in 20 μl reaction mixture with oligo-dT primers. Following the reverse transcription, PCR amplification of cDNA was performed with 1 μl of the above reaction mixture, which was used as the template in the GenAmp PCR System 9700 (Applied Biosystem).

The PCR amplifications of the chalcone synthase (CHS), chalcone isomerase (CHI), phenylalanine-ammonia lyase (PAL), isoflavone synthase (IFS) and actin-encoding sequences were performed with primers listed below in the 25 μl reactions carried out for 25 cycles with Allegro Taq Polymerase DNA (Novazym). The annealing temperatures were: 52 °C for the CHS and IFS, and 56 °C for the PAL and actin primers. The PCR products were analyzed using the agarose gel electrophoresis.

The sequences of the primers:

- CHS F 5′-ATCCTGATTTCTACTTCAGA-3′
- CHS R 5′-GGTGCCATATAAGCACAAA-3′
- PAL F 5′-CCAAGTCAATTGAGAGGGAG-3′
- PAL R 5′-CATCTTGGTTGTGCTGCTC-3′
- actin F 5′- GCATTGTTGGTCCTCCTCG-3′
- actin R 5′-TGTGCCTCATCCCCAACATA-3′
- IFSR 5′-CACAACAAGACCCTTGATT-3′
- IFSF 5′-GGACCTTACTGGAAGTTCAT-3′

### Data analysis

The analysis was done independently for the chosen secondary metabolites and was based on differences observed in LC-MS and/or GC-MS profiles registered between the treated and control plants. Baseline correction and alignment of all extracted mass peaks across all LC-MS analyses were done in MetAlign, developed by RIKILT (Institute of Food Safety, Wageningen University and Research Centre) and provided at www.metlign.wur.nl (Lommen 2009). Statistical analyses were carried out using the Microsoft Excel and MarkerLynx (Waters) was used for the GC-MS results.

## Results and discussion

### Characterization of *C. lupini* phytotoxic secondary metabolites

*Colletotrichum lupini* secondary metabolites extracted from the liquid culture medium were submitted to reversed-phase C18 flash column chromatography. In order to monitor phytotoxic fungal metabolites, the column eluate was spotted on leaves of *L. angustifolius* and *L. albus* plants. Fractions eluted with 55 % methanol caused the development of necrotic spots appearing on the leaf surface several hours after the application (Figure S1). The HPLC analysis of these fractions revealed the presence of multiple compounds with identical UV absorbance spectra consisting of a single maximum of absorbance at 270 nm. According to the HPLC-MS analysis, these compounds were characterized with different molecular weights, but similar fragmentation patterns (Figure S2). The high-resolution MS analysis helped establish the elemental composition of eight compounds, **A–H** present in the fraction, as shown in Table 1. It is noteworthy that at least some of these natural products were synthesized by the fungus in several isomeric forms that could be resolved using the HPLC (Figure S2). Compound **B** with the retention time of 8.54 min was the most abundant among the *C. lupini* metabolites and attempts were made to purify it using the preparative HPLC. However, the resulting sample (1.2 mg) was not sufficiently pure for obtaining a good quality NMR spectra required for the structural characterization. The fraction containing compounds **A–H** obtained after the flash column chromatography was used for the further experiments during plant elicitation with the fungal phytotoxin.

**Table 1:** Fraction of toxic natural products isolated from the culture medium of the fungus, *Colletotrichum lupini*—molecular formulas estimated from the *m/z* values of [M + H] ^+^ ions

Compound	Molecular formula	*m/z* of [M + H]^+^ ion		Error (ppm)
		Measured	Calculated	
A	C_19_H_35_NO_4_	342.2630	342.2639	2.73
B	C_19_H_33_NO_5_	356.2437	356.2431	−1.43
C	C_19_H_35_NO_5_	358.2588	358.2588	−0.05
D	C_21_H_37_NO_5_	384.2733	384.2744	2.99
E	C_21_H_39_NO_5_	386.2896	286.2901	1.21
F	C_21_H_35_NO_6_	398.2536	398.2537	0.22
G	C_21_H_37_NO_6_	400.2693	400.2694	0.04
H	C_23_H_39_NO_7_	442.2789	442.2799	2.36

### LC-MS identification of isoflavones and their glycoconjugates

Isoflavone and flavone glycoconjugates in leaves of *L. angustifolius* were studied earlier using HPLC-MS (Muth et al. 2008, 2009). Thirty-five flavone and isoflavone mono- and diglycosides partially acylated with malonic acid molecules were recognized in the lupin plant tissue then. Several of these compounds were isobaric or isomeric compounds characterized by a different substitution (hydroxylation and/or methoxylation) of the B ring of the aglycone moieties and the positions of the sugars and acyl groups. Moreover, various patterns of the glycosylation with different sugar substituents (hexoses, deoxyhexoses or pentoses) were observed. During the present research, additional pseudo-MS^3^ experiments were performed during the LC/MS analyses (Abranko et al. 2011). This methodical approach resulted in an unambiguous identification of the aglycones of the glycosylated isomeric natural products (Figure 3S). The application of the Advion Triversa Nanomate unit was especially efficient in the analysis of compounds present in minor amounts and additionally increased the number of the detected flavonoids (see Table 2). In particular, glycoconjugates of the isoflavones, luteone and wighteone, which were identified formerly in Mexican lupins (Stobiecki et al. 2010), but not recognized in the European species, were found during the described experiments. Mono- and diglucosides of these aglycones, some of those acylated with malonic acid molecules were recorded (Table 2). The number of detected flavonoid compounds increased twice—to 72 compounds listed in Table 2 as a result of all described improvements in the analytical approach.

**Table 2:** Flavonoid glycoconjugates and free aglycones detected in leaves of narrow leaf lupin (*L.* *angustifolius*)

Nr	Rt (min)	MW	Exact mass of [M + H]^+^ ion		Compound detected in *Lupinus* *angustifolius* leaves
			Calculated	Observed	
1	2.7	594	595.1657	595.1705	Genistein *C*-diglucoside
2	3.3	448	449.1078	449.1088	2′-hydroxygenistein 7-*O*-glucoside^a,b^
3	3.4	696	697.1611	697.1605	2′-hydroxygenistein diglucoside malonylated (I)^a,b^
4	3.5	432	433.1129	433.1122	Genistein 8-*C*-glucoside^b,c^
5	3.7	680	681.1661	681.1651	Genistein 4′,7di-*O*-glucoside malonylated (I)^a,b^
6	3.8	680	681.1661	681.1669	Genistein 4′,7di-*O*-glucoside malonylated (II)^a,b^
7	3.9	756	757.2186	757.2187	Chrysoeriol glucoside–xylosylglucoside^b^
8	3.9	782	783.1614	783.1615	2′-hydroxygenistein 4′,7di-*O*-glucoside dimalonylated^a^
9	4.0	610	611.1607	611.1610	Quercetin 3-*O*-rhamnosylglucoside^b^
10	4.2	518	519.1133	519.1136	Genistein 8-*C*-glucoside malonylated
11	4.2	464	465.1028	465.1015	Quercetin *O*-glucoside
12	4.3	448	449.1078	449.1061	Luteolin 7-*O*-glucoside^a,d^
13	4.3	518	519.1133	519.1138	Genistein *O*-glucoside malonylated^a^
14	4.3	680	681.1661	681.1661	Genistein *O*,*C*-diglucoside malonylated^a^
15	4.3	842	843.2190	843.2203	Chrysoeriol glucoside–xylosylglucoside malonylated (I)^b^
16	4.3	432	433.1129	433.1136	Genistein 7-*O*-glucoside^a,b,c,d^
17	4.3	534	535.1082	535.1075	2′-hydroxygenistein 7-*O*-glucoside malonylated (I)^b^
18	4.4	462	463.1235	463.1230	Chrysoeriol 8-*C*-glucoside^c^
19	4.4	842	843.2190	843.2183	Chrysoeriol glucoside–xylosylglucoside malonylated (II)^b^
20	4.4	680	681.1661	681.1667	Genistein diglucoside malonylated^b^
21	4.5	842	843.2190	843.2217	Chrysoeriol glucoside–xylosylglucoside malonylated (III)^b^
22	4.5	766	767.1665	767.1669	Genistein 4′,7di-*O*-glucoside dimalonylated (I)^a,b^
23	4.6	766	767.1665	767.1662	Genistein 4′,7di-*O*-glucoside dimalonylated (II)^a,b^
24	4.6	534	535.1082	535.1075	2′-hydroxygenistein 7-*O*-glucoside malonylated (II)^a,b^
25	4.6	462	463.1235	463.1230	Chrysoeriol *O*-glucoside (I)^b^
26	4.6	448	449.1078	449.1088	Kaempferol-3-*O*-glucoside^a,d^
27	4.7	842	843.2190	843.2205	Chrysoeriol glucoside–xylosylglucoside malonylated (IV)^b^
28	4.8	564	565.1552	565.1551	Apigenin *O*-xylosylglucoside^a^
29	4.8	518	519.1133	519.1142	Genistein *C*-glucoside malonylated
30	4.8	782	783.1614	783.1618	2′-hydroxygenistein 7-*O*-diglucoside dimalonylated
31	4.8	928	929.2194	929.2209	Chrysoeriol glucoside–xylosylglucoside dimalonylated (I)^b^
32	4.9	928	929.2194	929.2201	Chrysoeriol glucoside–xylosylglucoside dimalonylated (II)^b^
33	4.9	432	433.1129	433.1131	Apigenin 7-*O*-glucoside^a,c,d^
34	5.0	928	929.2194	929.2192	Chrysoeriol glucoside–xylosylglucoside dimalonylated (III)^b^
35	5.0	766	767.1665	767.1662	Apigenin 4′,7di-*O*-glucoside dimalonylated^a^
36	5.0	696	697.1611	697.1615	2′-hydroxygenistein 7-*O*-diglucoside malonylated (II)
37	5.1	666	667.1505	667.1512	Luteolin xylosylglucoside malonylated^a^
38	5.1	594	595.1657	595.1665	Chrysoeriol *O*-xylosylglucoside^b^
39	5.1	548	549.1239	549.1245	Chrysoeriol *C*-glucoside malonylated
40	5.2	534	535.1082	535.1089	Kaempferol-*O*-glucoside malonylated^a^
41	5.2	518	519.1133	519.1124	Genistein 7-*O*-glucoside malonylated (I)^a^
42	5.3	650	651.1556	651.1549	Genistein 7-*O*-xylosylglucoside malonylated (I)
43	5.3	462	463.1235	463.1234	Chrysoeriol *O*-glucoside (II)
44	5.4	518	519.1133	519.1131	Genistein 7-*O*-glucoside malonylated (II)^a,b^
45	5.4	678	679.2233	679.2215	Luteone *O*-diglucoside
46	5.5	650	651.1556	651.1551	Genistein 7-*O*-xylosylglucoside malonylated (II)^b^
47	5.5	680	681.1661	681.1678	Chrysoeriol *O*-xylosylglucoside malonylated (I)^b^
48	5.6	518	519.1133	519.1139	Apigenin *O*-glucoside malonylated (I)^a^
49	5.6	650	651.1556	651.1553	Apigenin *O*-xylosylglucoside malonylated (I)^a^
50	5.7	680	681.1661	681.1678	Chrysoeriol *O*-xylosylglucoside malonylated (II)^b^
51	5.8	518	519.1133	519.1136	Apigenin *O*-glucoside malonylated (II)^a^
52	5.8	680	681.1661	681.1681	Chrysoeriol *O*-xylosylglucoside malonylated (III)^b^
53	5.9	286	287.0550	287.0547	2′-hydroksygenistein^a,b,c,d^
54	5.9	548	549.1239	549.1253	Chrysoeriol *O*-glucoside malonylated^b^
55	5.9	662	663.2283	663.2269	Wighteone *O*-diglucoside
56	6.0	680	681.1661	681.1665	Chrysoeriol *O*-xylosylglucoside malonylated (IV)^b^
57	6.1	766	767.1665	767.1660	Chrysoeriol *O*-xylosylglucoside dimalonylated (I)^b^
58	6.2	766	767.1665	767.1683	Chrysoeriol *O*-xylosylglucoside dimalonylated (II)^b^
59	6.2	766	767.1665	767.1776	Chrysoeriol *O*-xylosylglucoside dimalonylated (III)^b^
60	6.5	850	851.2240	851.2225	Luteone *O*-diglucoside dimalonylated
61	6.8	748	749.2287	749.2290	Wighteone *O*-diglucoside malonylated
62	6.9	516	517.1704	517.1701	Luteone 7-*O*-glucoside
63	5.9	834	835.2291	835.2285	Wighteone *O*-diglucoside dimalonylated
64	7.0	764	765.2237	765.2241	Luteone *O*-diglucoside malonylated (II)
65	7.1	270	271.0601	271.0602	Genistein^a,b,c,d^
66	7.2	270	271.0601	271.0603	Apigenin ^a,c,d^
67	7.8	603	603.1708	603.1713	Luteone 7-*O*-glucoside malonylated
68	8.5	500	501.1755	501.1749	Wighteone 7-*O*-glucoside
69	9.8	586	587.1759	587.1767	Wighteone 7-*O*-glucoside malonylated
70	7.5	300	301.0707	301.0714	Chrysoeriol^a,c,d^
71	14.4	354	355.1176	355.1178	Luteone^a,b,c,d^
72	14.6	338	339.1227	339.1231	Wighteone^a,b,c,d^

^a^Identification of aglycone based on mass spectra registered in MS^3^ mode

^b^Compounds reported in earlier published papers: Muth et al. (2008; 2009)

^c^PubChem ID numbers of the identified compounds: genistein 8-C-glucoside—44257270; genistein 7-*O*-glucoside—44257273; chrysoeriol 8-*C*-glucoside—44258170; apigenin 7-*O*-glucoside—5280704; 2′-hydroksygenistein—5282074; genistein—5281377; apigenin—5280443; chrysoeriol—5280666; luteone—5281797; wighteone—5281814

^d^Identification of compound based on comparison with standard

The pattern of acylation of the flavone and isoflavone glycosides with malonic acid was an important factor that had a major impact on the structural variability of the studied compounds. An elucidation of the malonic group placement on the sugar moieties was not possible only on the basis of mass spectra registered during the LC-MS analyses, so a purification of the compounds and the NMR analyses would be necessary. The application of a rapid-resolution LC system assisted with the additional fractionation of the LC column eluate using the TriVersa device permitted an efficient separation of isomers of malonylated isoflavone, flavone and flavonol glycosides, but still no definite conclusions regarding the substitution pattern of these isomeric glycoconjugates with malonyl groups could be drawn. Nevertheless, the possibility to use the off-line mode of obtaining MS spectra using TriVersa, especially in MS^2^ or pseudo-MS^3^ mode, provided better quality spectra. Profiles of the target flavonoids and their conjugates present in the studied samples, including quantitative changes of positional isomers of malonylated flavonoid glycoconjugates, were elucidated on the basis of the single-ion chromatograms of protonated molecules registered in the MS mode.

### GC-MS identification of natural products

**Fig. 1:**
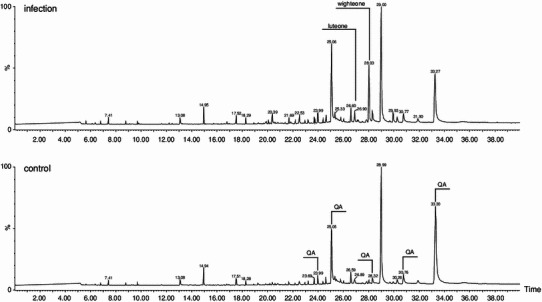
GC-MS total ion chromatogram registered for cuticle and wax washings of the lupin plantlets (cv. Mirela–bitter) after infection with *C. lupini* spores (*top*) and control plants (*bottom*). Analyses were performed for two biological samples at two technical repetitions for each sample. The identified QA are listed in Table 1S

**Fig. 2:**
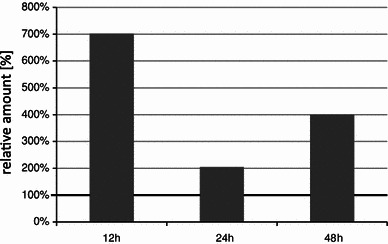
Contents of wighteone detected in the samples obtained by washing of lupin leaves with CH_2_Cl_2_, obtained at three time points after elicitation (12, 24, and 48 h) of lupin plants (*L. angustifolius* cv. Sonet) with phytotoxin obtained from cultures of fungus *C. lupini*. The GC-MS measured amounts of wighteone are expressed in relation to that in the control plants (= 100 %). Analyses were performed for two biological samples at two technical repetitions for each sample

Compounds present on the surface of lupin leaves in the wax and suberin layer were analyzed using the GC-MS. Samples were prepared from the control plants as well as from the ones subjected to the infection with the *C. lupini* spores and/or elicitation with phytotoxic metabolites of the fungus sprayed on the plantlets. The cuticle components, such as waxes and secondary metabolites, were washed with methylene chloride from leaves of two high alkaloid (“bitter”) and two low alkaloid (“sweet”) *L. angustifolius* cultivars. Conditions of this extraction procedure (organic solvent and time of washing) were optimized in order to achieve the maximal yield and avoid a damage of cell walls and membranes causing a leakage of cytoplasm components. The samples were derivatized with trimethylsilyl (TMS) groups to block the polar groups of the molecules and increase the volatility of the studied compounds injected on the GC column (see ‘Materials and methods’). Different classes of primary and secondary metabolites: alkaloids, fatty acids and their esters, sterols and isoflavones were recognized in the studied samples on the basis of comparison of the registered mass spectra with the data bases. The most abundant and important group of natural products detected on the lupin leaf surface were the quinolizidine alkaloids (QA). To no astonishment, a substantial quantitative difference in the alkaloid contents (two orders of magnitude) was observed between the sweet and bitter cultivars studied. The 14 detected alkaloids were eluted from the GC column in a wide range of RT due to the presence of the free bases (angustifolin, lupanin and multiflorin) as well as 13-hydroxylupanin esterified with various organic acids (see Table 1S). It is noteworthy that the total amount of alkaloids present in the bitter lupin cultivars was dominated by the 13-hydroxylupanin esters, whereas the non-esterified QA were more abundant in the sweet cultivars. The individual QAs were identified on the basis of their RT and the respective mass spectra in comparison with these obtained for the standard compounds. There were no quantitative differences observed in abundances of QAs in samples prepared from control, *C. lupini* infected or phytotoxin-elicited plants (data not presented). Among the flavonoid compounds only prenylated isoflavones wighteone and luteone that are known lupin phytoalexins (Ingham et al. 1983) were also detected on the leaf surface of plants infected with the *C. lupini* spores or elicited with its phytotoxic metabolites (Fig. 1). Amounts of wighteone and luteone detected on the leaf surface were much smaller than these of QAs, especially in the case of the bitter cultivars (Mirela and Karo). There was a notable difference between the prenylated isoflavone aglycones found on leaf surface of the infected and elicited plants. While luteone and wighteone were recognized in the former group, only wighteone could be detected on the latter plants. A large increase of the amount of free wighteone was observed on the leaf surface of the phytotoxin-elicited plants in relation to the control as early as 12 h after the treatment (Fig. 2) when it was 7 times higher and it remained at the elevated level (400 % of the control) after 48 h. It should be noted that neither infection with the fungal spores nor phytotoxin treatment caused any change in presence of flavonoid aglycones other than the prenylated isoflavones on the surface of *L. angustifolius* leaves. Similarly, no changes in fatty acid and sterols were observed as a result of the plant treatment.

### PCR analysis of phenylpropanoid pathway genes

Changes in transcription of genes responsible for the key enzymes participating in the isoflavone biosynthesis pathway (PAL, CHS, CHI and IFS) in response to infection with *C. lupini* and treatment with the phytotoxic metabolites of the fungus were studied at different time points. The transcription of the studied genes was monitored after 24 and 48 h in the case of spraying plants with the phytotoxin solution and, additionally, 96 and 168 h after infection with the *C. lupini* spores. The level of transcription of three genes (CHS, CHI and IFS) under study increased in the treated plants above the level in the control as a result of both types of plant treatment (Figure 4S), whereas the expression of PAL as well as the positive control actin genes remained on the same level. Additionally, it was found that the transcription of the monitored enzymes was higher in the leaves submitted to the phytotoxin deposition than in the untreated leaves collected from the treated plants (data not presented). On the basis of these data, we can conclude that the regulatory mechanisms of the lupin plant response activated during their interaction with spores and fungal toxin involve induction of the isoflavone biosynthesis pathway.

### Comparison of profiles of isoflavone glycoconjugates and free aglycones in tissues of lupin plants elicited with toxin or infected with spores

**Fig. 3:**
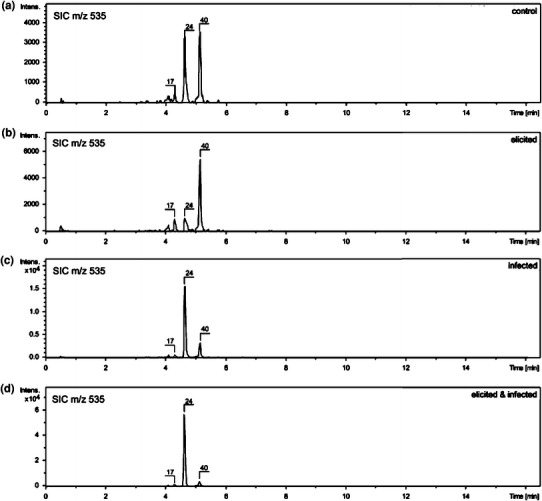
Single-ion chromatograms (*SIC*) registered for [M + H]^+^ ions at *m/z* 535 for malonylated 2′-hydroxygenistein-*O*-glucosides (**17**, **24**) and their isobaric compound, malonylated kaempferol-*O*-glucoside (**40**) in samples obtained from leaves of sweet cultivar of narrow leaf lupin *L. angustifolius* (cv. Sonet). **a** Control, **b** elicited, **c** infected with spores and **d** elicited and after 48 h infected with fungal spores lupin seedlings. All samples of plant material were collected 7 days after infection

As it was mentioned previously, different mass spectrometric methods were elaborated for the structural characterization of the studied flavonoid glycoconjugates and for their quantitative analysis. These methods differed in settings of the ISCID (in source collision-induced dissociation) parameters that influenced the relative intensity of the [M + H]^+^ ions and the fragment ions. While the structural elucidation was based mainly on MS^2^ and pseudo-MS^3^ experiments, the quantitative analysis was performed in conditions, in which intense [M + H]^+^ ions were registered. Anyway, a discussion of a biological role of the natural products present in the analyzed samples demands their proper identification. For example, two isomers of malonylated glucosides of 2′-hydroxygenistein (compounds **17** and **24** with RT of 4.3 and 4.6 min, Table 2; Fig. 3) and malonylated kaempferol glucoside (compound **40**, RT of 5.2 min) were isobaric compounds with a molecular weight of 534 Da as identified in the analyzed samples. The abundance of only one 2′-hydroxygenistein derivative (compound **24**) was changed substantially as a result of infection or elicitation of lupin plants (Fig. 3).

**Fig. 4:**
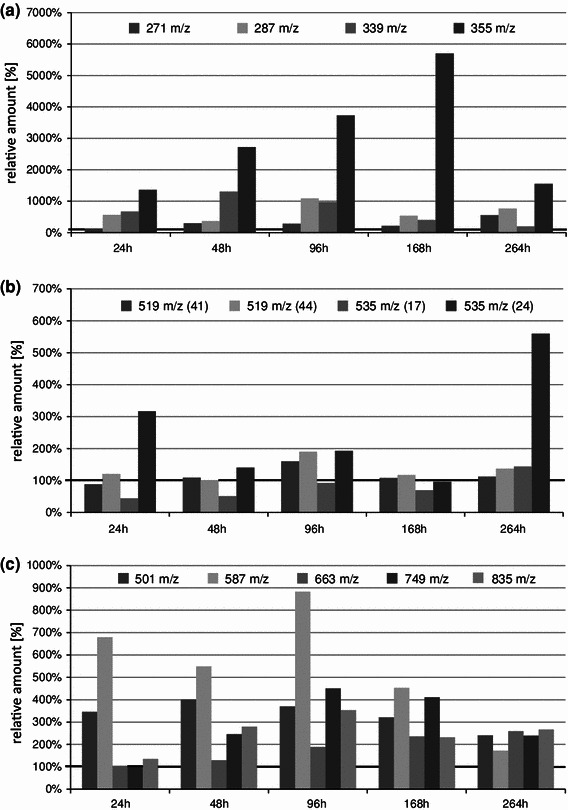
Contents of chosen isoflavones and their glycoconjugates detected after infection with *C. lupini* spores of lupin plants (*L. angustifolius* cv. Sonet), expressed in relation to the control (=100 %), time points: 24, 48, 96, 168 and 264 h after infection. Free isoflavone aglycones: genistein—[M + H]^+^ at *m/z* 271 (**65**), 2′-hydroxygenistein—[M + H]^+^ at *m/z* 287 (**53**), wighteone—[M + H]^+^ at *m/z* 339 (**72**), luteone—[M + H]^+^ at *m/z* 355 (**71**) (**a)**; isoflavone glycoconjugates (malonylated genistein-7-*O*-glucoside—[M + H]^+^ at *m/z* 519—two isomers (**41**, **44**), malonylated 2′-hydroxygenistein-7-*O*-glucoside—[M + H]^+^ at *m/z* 535—two isomers (**17**, **24**) (**b)**; and wighteone glyconjugates: wighteone glucoside—[M + H]^+^ at *m/z* 501 (**68**), malonylated wighteone glucoside—[M + H]^+^ at *m/z* 587 (**69**), wighteone diglucoside—[M + H]^+^ at *m/z* 663 (**55**), malonylated wighteone diglucoside—[M + H]^+^ at *m/z* 749 (**61**) and dimalonylated wighteone diglucoside—[M + H]^+^ at *m/z* 835) (**63**) (**c**). Plant material was collected at five time points after infection: 24, 48, 96, 168 and 264 h. The values are averages from LC-MS analyses of three biological samples with two technical repetitions for each one

**Fig. 5:**
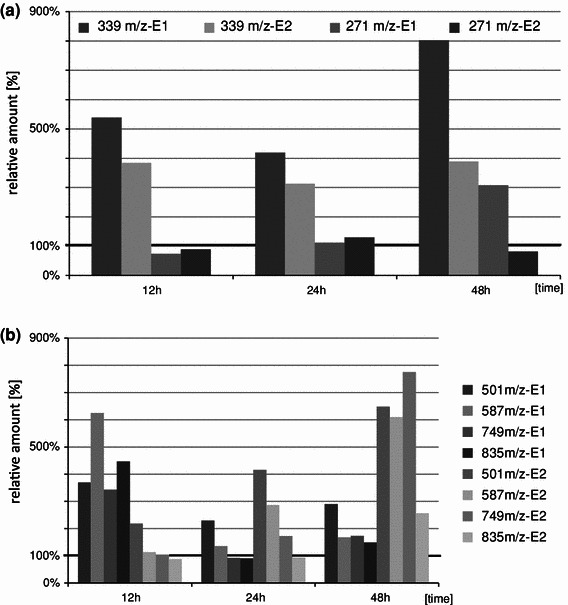
Contents of wighteone ([M + H]^+^ at *m/z* 339—**72**) and genistein ([M + H]^+^ at *m/z* 271—**65**) (**a**) and wighteone glycoconjugates (wighteone glucoside [M + H] + at *m/z* 501 (**68**), malonylated wighteone glycoside—[M + H]^+^ at *m/z* 587 (**69**), malonylated wighteone diglycoside—[M + H]^+^ at *m/z* 749 (**61**) and dimalonylated wighteone diglycoside—[M + H]^+^ at *m/z* 835) (**63**) (**b)**. Natural products detected after elicitation of lupin plants (*L. angustifolius* cv. Sonet) with the fungal toxin expressed in relation to the control (= 100 %). Elicitation of lupin plants by deposition of the toxin on leaves (point infection)—E_1_; spraying of the toxin on leaves—E_2_. Plant material was collected at three time points after elicitation: 12, 24 and 48 h. The values are averages from LC-MS analyses of three biological samples with two technical repetitions for each one

**Fig. 6:**
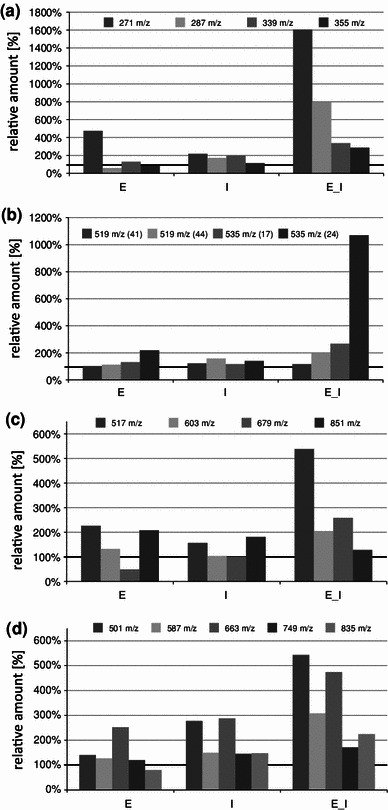
Contents of chosen isoflavones and their glycoconjugates detected in lupin seedlings of 2 weeks old after elicitation (*E*), infection with *C. lupini* spores (*I*) or elicitation followed with infection 48 h after elicitation of lupin plants (*E* *+* *I*) (*L. angustifolius* cv. Sonet), expressed in relation to the control (= 100 %). On the graphs are presented: relative amounts of free aglycones—genistein—[M + H]^+^ at *m/z* 271 (**65**), 2′-hydroxygenistein—[M + H]^+^ at *m/z* 287 (**53**), wighteone—[M + H]^+^ at *m/z* 339 (**72**), luteone—[M + H]^+^ at *m/z* 355 (**71**) (**a**); isomers of malonylated glycosides of genistein and 2′-hydroxygenistein: (malonylated genistein-7-*O*-glycoside—[M + H]^+^ at *m/z* 519—two isomers (**41**, **44**), malonylated 2′-hydroxygenistein-7-*O*-glycoside—[M + H]^+^ at *m/z* 535—two isomers (**17**, **24**) (**b)**; luteone glucoside—[M + H]^+^ at *m/z* 517 (**62**), malonylated luteone glucoside—[M + H]^+^ at *m/z* 603 (**67**), luteone diglucoside—[M + H]^+^ at *m/z* 679 (**45**) and dimalonylated luteone diglucoside—[M + H]^+^ at *m/z* 851 (**60**) (**c)** and wighteone glyconjugates: wighteone glucoside—[M + H]^+^ at *m/z* 501 (**68**), malonylated wighteone glycoside—[M + H]^+^ at *m/z* 587 (**69**), and wighteone diglycoside—[M + H]^+^ at *m/z* 663 (**55**), malonylated wighteone diglycoside—[M + H]^+^ at *m/z* 749 (**61**) and dimalonylated wighteone diglycoside—[M + H]^+^ at *m/z* 835) (**63**) (**d**). Control and treated plants were collected at the same time (9 days from the start of elicitation)

Three different types of plant treatment were applied during our experiments, in which changes to the contents of isoflavones and their glycoconjugates in tissues of *L. angustifolius* leaves were studied. Plants were (1) sprayed with the *C. lupini* spore suspension, (2) elicited with the fungus phytotoxic compounds applied either on wounded leaves or sprayed on plants and (3) sprayed with the phytotoxins and with spores 48 h later. Changes of the studied isoflavonoid profiles were detected as a result of all the treatments applied; however, the plant responses were different and occurred after different times depending on the way of their treatment. Amount of each compound in the studied tissue was calculated using the total intensity of the respective [M + H]^+^ ion in relation to that of the internal standard (luteolin) and the mass of the sample. The mean values obtained from three biological repetitions, each measured in two chromatographic runs are presented in the Supplementary Materials (Figures 5S–8S). Figures 2, 4, 5 and 6 show the same data as the measured amount of the studied compound in leaves of plants treated with fungal compounds or infected with the *C. lupini* spores in relation to that detected in the control plants.

In the case of infection with spores of *C. lupini*, a maximum response directed to the synthesis of phenolic secondary metabolites was recorded after 7 days (168 h), but the onset of the increased accumulation of these compounds was observed after 24 h (Fig. 4). Three free isoflavone aglycones (2′-hydroxygenistein and two known phytoalexins, wighteone and luteone) were found in tissues in much increased amounts at all studied time points in comparison to these observed in the control plants. In contrast, the amount of genistein was elevated substantially at the last time point of the experiment. In particular, the levels of free luteone observed in leaves of the infected plants after 7 days were more than 50 times higher than these in the control plants. It should be noted that the contents of some isoflavonoid glycoconjugates also increased. Interestingly, only one isomeric malonylated 2′-hydroxygenistein glucoside (compound **24**) was observed in an increased amount, whereas another isomer (compound **17**) remained on the level observed in the control. Moreover, levels of different wighteone glycoconjugates were also increased shortly after the infection (Fig. 4c) and their amount decreased at the later time points.

Rapid and substantial changes in profiles of isoflavones and their derivatives were observed in the experiments, in which the fungal phytotoxin was applied as an elicitor of the plant response (Fig. 5). Several interesting differences were noted in the reaction of the phytotoxin-elicited plants in comparison to the infected ones. Spraying of plants with phytotoxins caused almost a fourfold increase of the amount of the free wighteone above the levels in the control plants just 12 h after the “elicitation 2” and this was observed until 48 h (Fig. 5a). The wighteone level was even higher in plants, in which phytotoxin was deposited on wounded leaves (“elicitation 1”, up to 800 % of the control), but some of this should be attributed to the effect of wounding. In the “elicitation 1”, the wighteone level increase was observed only in the treated leaves and no SAR-like effect was observed in the untreated leaves of the elicited plants. The “elicitation 1” did not cause any induction of luteone, the other prenylated isoflavone phytoalexin. Some differences between both types of elicitation “E1 and E2)” effects could be observed in the accumulation of the wighteone derivatives: malonylated and dimalonylated diglucosides (compounds **61** and **63**, Table 2) and monoglucoside and monoglucoside malonylated (compounds **68** and **69**). Amounts of these compounds increased within 12 h after the “elicitation E1” to 350–600 % of the level in the control plants and then gradually decreased until 48 h after the treatment. On the other hand, the increase of their amount was slower as a result of the “elicitation E2”, but even 800 % of the control level was reached after 48 h (Fig. 5b).

Accumulation of isoflavone derivatives in response to the elicitation with the phytotoxic metabolites of *C. lupini* and subsequent infection with spores of the fungus was observed in the third described experiment. Plants sprayed with the phytotoxic metabolites were infected after 48 h and leaves thereof were collected 7 days (168 h) later. Contents of isoflavones in control plants, plants either only elicited or only infected and plants both elicited and infected were compared at the same time, 9 days (216 h) after the start of the experiment. This time was selected as a result of the earlier experiments, in which the highest induction of the metabolite accumulation was observed between 96 and 168 h after the infection. Genistein and 2′-hydroxygenistein were the most abundant free aglycones in leaves of plants infected after the previous elicitation and their contents increased to a level 1600 % higher than those of the control (Fig. 6). Both these isoflavone aglycones were induced to some extent as a result of elicitation or infection performed independently, but the induction observed in plants infected 48 h after the elicitation was much more pronounced. Similar observation could be made for some specific glycoconjugates of 2′-hydroxygenistein, luteone and wighteone (Fig. 6b). On the other hand, contents of numerous compounds was either not changed or only slightly increased as an effect of the applied treatments of plants.

Analysis of plant reactions during pathogenic microorganism attack or elicitation with various high- or low-molecular-weight natural products may indicate that different reactions at various molecular levels (RNAs, proteins and secondary metabolites) are developed in the studied plants. These reactions may differ in various plants due to numerous defense mechanisms developed by plants during their evolution at the perception (MAMP) and recognition (PRR) levels of the natural products signaling the infection by phytopathogens. That is why the response of narrow leaf lupin plants to the infection with the anthracnose fungus spores and to the elicitation with the defined effectors may involve expression of various proteins and further synthesis of different secondary metabolites, due to activation of different enzymes in the metabolic pathways. Induction of the expression of genes involved in the isoflavone biosynthesis pathway as well as increase of the contents of the target natural products, either in the form of the free aglycones or as their glycoconjugates in plants of *L. angustifolius* infected with *C. lupini* fungus, was studied in the experiments described herein. Prenylated isoflavones are known components of lupin defense system. Phytoalexins wighteone and luteone are synthesized by lupin plants as a result of fungal infection (Ingham et al. 1983) and licoisoflavones A and B present in *L. angustifolius* roots have been shown to deter larvae of pasture scarabs *Costelytra zealandica* and *Heteronychus arator* (Lane et al. 1987). It was previously found (Muth et al. 2009) that wighteone and luteone were accumulated differently in leaves of plants infected with the *C. lupini* fungus. This observation has been currently supported by finding that only wighteone was found in increased amounts on the surface as well as within leaf tissues of plants that were treated with phytotoxins of the fungus, whereas both wighteone and luteone were induced by the infection.

Treatment of some plant species with low-molecular-weight signaling compounds, like salicylic acid or methyl jasmonate (the well-known elicitors of response to biotic stresses), may induce systemic acquired resistance (SAR), as it was observed in the case of *M. truncatula* (Naoumkina et al. 2007; Farag et al. 2008, 2009). However, SAR was not observed in the case of the presently studied deposition of the *C. lupini* phytotoxic compounds on leaves of *L. angustifolius* plantlets. On the other hand, the elicitation of lupin plants with these compounds prior to the application of fungal spores caused a much faster and more intense answer of such treated plants than it was observed after only the infection with the spores. An increased synthesis of certain malonylated forms of the isoflavone glycosides was one of the effects observed in the lupin plant response to fungal infection or treatment with fungal phytotoxic compounds. Similar effects were also observed for other plant species from the Fabaceae family that are subjected to biotic or abiotic stress (Lozovaya et al. 2004; Farag et al. 2008, 2009; Jasiński et al. 2009). Isoflavone *O*-malonyltransferases were recently characterized in *Medicago truncatula* and it was observed that the expression of these genes was stress-inducible (Zhao et al. 2011). However, these genes present in different legume plants do not exhibit sequence similiarities (Yu et al. 2008), and monitoring of malonyltransferases in lupin was not yet realized. It would be interesting to define the role of the acylation of the studied isoflavones in the stress conditions. At present, it is suggested that the malonylation of flavonoid glycosides plays an important role in intracellular transport of these compounds and the malonylated entities are recognized by MATE transporters and transferred to proper locations (vacuoles) in plant cells (Zhao et al. 2011).

## Concluding remarks

Many of the isoflavone glycoconjugates occur in the *L. angustifolius* leaves in several isomeric forms. These forms differ in the prenylation, glycosylation and/or malonylation pattern and contents of only some of them are changed as a result of infection. From the registered results, we can conclude that especially the malonylation of the glycosylated precursor of antibiotic natural products may play an important role. This may be exemplified by the registration of the increase of contents of only one out of two isomeric malonylated 2′hydroxygenistein glucosides. Generally, it is likely that the changes of only certain isoflavonoids contents and not the overall amount of these compounds are relevant in the plant response to fungal infection. There are major differences in the synthesis of secondary metabolites by lupin plants in response to fungal infection and to treatment with fungal phytotoxic compounds. Spraying lupin plants with toxins accelerates and strengthens their response at the level of synthesis of isoflavone phytoalexins or their precursors. While the first changes in the isoflavone profiles may be observed as early as 12 h after the phytotoxin deposition on the leaf surface, the altered composition of this pool of secondary metabolites persists in the infected plants for at least several days. In this situation, we can conclude that the perception of MAMPs at the level of low-molecular-weight natural products through the PRRs situated on the cell surface causes changes in the synthesis of plant secondary metabolites that play a role in the protection against fungal infection.

## Electronic supplementary material

Below is the link to the electronic supplementary material.

Supplementary material 1 (DOCX 16 kb)

Supplementary material 2 (PDF 888 kb)
