# Supplementary material for: Changes of phenolic secondary metabolite profiles in the reaction of narrow leaf lupin (Lupinus angustifolius) plants to infections with Colletotrichum lupini fungus or treatment with its toxin
Source: Metabolomics. 2012 Oct 30;9(3):575–89. doi: 10.1007/s11306-012-0475-8 (PMC3651525; doi:10.1007/s11306-012-0475-8)
Supplement: Supplementary file 1 — Supplementary material 1 (DOCX 16 kb) [file 11306_2012_475_MOESM1_ESM.docx]

## Legend to the Supporting Information

Laboratory procedures for extraction and analysis of phenolic secondary metabolites

*Extraction of phenolic secondary metabolites from plant tissues*

*Gas chromatography/mass spectrometry*

*Liquid chromatography/mass spectrometry*

Table 1S. Quinolizidine alkaloids identified in lupin leaves of *L. angustifolius* (cv. Mirela).

Figure 1S. Phytotoxicity of *Colletotrichum lupini* metabolites that were purified using reversed-phase C18 flash chromatography. The column eluate was spotted on needle punctured leaves of *L. angustifolius* (top) and *L. albus* (bottom).

Figure 2S. HPLC-MSn analysis of the phytotoxic fraction of the *Colletotrichum lupini* metabolites. Chromatograms were obtained in the positive ion mode and drawn for *m/z* A: 356; B: 358; C: 342; D: 382 and E: 400. The shown sequential MS spectra correspond to the [M+H]^+^ ion at *m/*z 358.

Figure 3S. Collision-induced mass spectra of flavonoid positional isomers genistein-7-*O-*glucoside [a]; and apigenin-7-*O*-glucoside [b] recorded in the MS2 and pseudo-MS3 modes for the positive [M+H]^+^ (at *m/z* 433) and negative [M-H]^-^ (at *m/z* 431) ions and the respective aglycone ions at *m/z* 271 and 269.

Figure 4S. Agarose gel electrophoresis of cDNAs corresponding to the transcripts of [A] chalcone isomerase (CHI); [B] isoflavone synthase (IFS) [C] chalcone synthase (CHS) and phenylalanine-ammonia lyase (PAL) genes and [D] the positive control – the actin gene.

Figures 5S-8S. Contents of different isoflavone aglycones and glycoconjugates in leaves of narrow leaf lupin (*L. angustifolius*) control plants and plants infected with *Colletotrichum lupini* spores or treated with fungal phytotoxic compounds. These data are shown in Figures 2 and 4-6 as relative changes of compounds amounts in result of the treatment.
