# Supplementary material for: Changes of phenolic secondary metabolite profiles in the reaction of narrow leaf lupin (Lupinus angustifolius) plants to infections with Colletotrichum lupini fungus or treatment with its toxin
Source: Metabolomics. 2012 Oct 30;9(3):575–89. doi: 10.1007/s11306-012-0475-8 (PMC3651525; doi:10.1007/s11306-012-0475-8)
Supplement: Supplementary file 2 — Supplementary material 2 (PDF 888 kb) [file 11306_2012_475_MOESM2_ESM.pdf]

Supporting Materials to the paper

Changes of phenolic secondary metabolite profiles in the reaction of narrow leaf lupin (*Lupinus angustifolius*) plants to infections with *Colletotrichum lupini* fungus or treatment with its toxin

by

Anna Staszków, Dorota Muth, Dorota Narożna, Cezary Mądrzak, Maciej Stobiecki*, Piotr Kachlicki*

Extraction of phenolic secondary metabolites from plant tissues

Extractions of phenolic secondary metabolites from green parts of the lupin plants were done in two independent ways. For the LC-MS analysis, the frozen leaves (100 mg fresh weight) were homogenized in 2 ml of 80% methanol (ball mill MM200, Retsch, Haan, Germany), and the suspension was placed in an ultrasonic bath for 30 min. Luteolin was added to the homogenates as an internal standard. The extracts were centrifuged and the supernatants were transferred to new screw-cupped tubes. The solvent was removed in a vacuum concentrator at room temperature (Savant SPD 121P, Thermo Electron Corporation, Waltham, USA). Samples were dissolved in 300 µl of 80% methanol in water and centrifuged at 10,000 rpm for 10 min, transferred to autosampler vials and immediately subjected to LC-MS analyses.

For GC-MS analysis of compounds present on lupin leaf surface, green parts of lupin plants (five plants for each sample) were washed with 100 ml of CH_2Cl_2 for 20 sec. The washing time was optimized to avoid damage of the cells, causing a leakage of cytosolic compounds that occurred with a prolonged action of the organic solvent. Collection of surface compounds was done at different time points after elicitation or infection. The obtained solutions were evaporated, then the sample was dissolved in 2 ml of CH_2Cl_2 and the volume corresponding to 2 mg of the original dry weight from the each sample was transferred to the Teflon-lined screw-capped vials and taken for further GC-MS analysis. Rybitol (20 µl of methanol solution at a concentration of 1 mg/ml) was added to each sample as an internal standard and a two-stage chemical derivatization procedure was performed. Forty µl of *O*-methylhydroxylamine hydrochloride solution in pyridine (20 mg/mL) was added to the sample and heated at 40°C for 90 min followed by addition of 70 µl MSTFA (*N*-acetyl-*N*-(trimethylsilyl)-trifluoroacetamide) and heating at 37°C for 30 min. The sample was centrifuged at 10,000 rpm for 10 min, transferred to autosampler glass vials and subjected to the GC-MS analyses. Two biological samples were collected from each object and two independent extracts were prepared and analyzed for each sample.

Gas chromatography/mass spectrometry

GC-MS analyses of leaf surface compounds were performed with Agilent 6890 N gas chromatograph with a 7683 autosampler (Agilent Technologies, Stockport, UK) equipped with a DB-5 column (30 m × 0.25 mm i.d., film thickness 0.25 µm) from J&W Scientific Co. (USA) and coupled to the time-of-flight mass spectroscope (MS-ToF) analyzer from Waters. Helium was used as the carrier gas at a flow rate of 1 ml/min. The GC oven temperature program was as follows: 2 min at 70°C, raised by 10°C min^{-1} to 300°C, and held for 15 min at 300°C. The total analysis time was 45 minutes. The injector temperature was 250°C and 50% of the recovered vapor was passed into the chromatography column (split 50). The interface temperature was 230°C and source temperature was 250°C. In-source fragmentation was performed with 70 eV energy. Mass spectra were recorded in the 50-650 *m/z* range and data were analyzed using the Waters MassLynx ver. 4.1.

Liquid chromatography/mass spectrometry

LC-MS analyses of plant extracts were performed with an Agilent (Waldbronn, Germany) RR 1200 system (binary pump SL, diode array detector G1315C Starlight and a G1367C SL automatic injector) connected to a micrOToF-Q mass spectrometer from Bruker Daltonics (Bremen, Germany) and/or the TriVersa NanoMate system (Advion, USA).

Analyses were carried out using Zorbax Eclipse XDB C18 or Poroshell 120 EC-C18 columns (2.1×100 mm, granulation of 1.8 µm, Agilent). Chromatographic separation was performed at a 0.6 ml/min flow rate using mixtures of two solvents: A (99.5% H_2O/0.5% formic acid v/v) and B (99.5% acetonitrile/0.5% formic acid v/v) with a 3:2 split of the column effluent, so 0.2 ml/min was delivered to the ESI ion source. The elution steps were as follows: 0–5 min linear gradient from 10 to 30% of B, 5–12 min isocratic at 30% of B, 12–13 min linear gradient from 30–95% of B, and 13–15 min isocratic at 95% of B. After returning back to the initial conditions, the equilibration was achieved after 4 min.

The ESI source of the micrOToF-Q mass spectrometer operated at a voltage of ±4.5 kV, ion transfer energy of 7 eV during the MS or MS^2 experiments. Nitrogen nebulization was performed at 1.2 bar and a dry gas flow of 8.0 l/min at temperature of 220°C. The instrument was operated using the micrOTOF control program ver. 2.3 and data were analyzed using the Bruker Data Analysis ver. 4 package. The system was calibrated externally using the calibration mixture containing sodium formate clusters. Additional internal calibration was performed for every run by injection of the calibration mixture using the diverter valve during the LC separation. All calculations of *m/z* values were done with the HPC quadratic algorithm with the accuracy of at least 5 ppm.

Isoflavonoid conjugates were identified by comparison of the exact molecular masses and fragmentation pathways of precursor $[M+H]^+$ ions recorded during CID MS/MS experiments with those obtained for standards and data from the literature. The targeted MS/MS experiments were performed using a collision energy ranging from 15 to 30 eV (positive ion mode), depending on the molecular masses of compounds. Pseudo-MS^3 experiments at an increased ionization potential of 20-25 eV were also performed to achieve high fragmentation of protonated molecules $[M+H]^+$. The ionization energy (in source collision-induced dissociation – ISCID) was changed to 80 eV or 85 eV in positive and negative ion modes, respectively, for the pseudo-MS3 experiments. The instrument was operated at a resolution higher than 15 000 FWHM (full width at half maximum). The spectra were recorded in the targeted mode within the *m/z* range of 50-1000 and metabolite profiles were registered in the positive ion mode. For identification of the compounds, the instrument was operated in the CID MS/MS mode and single-ion chromatograms for exact masses of $[M+H]^+$ ions (±0.005 Da) were recorded.

For more effective separation and identification of isomeric flavonoids compounds, a chip-based nano-electrospray ionization source (TriVersa NanoMate system) in the LC-MS Fraction Collection mode was used.

Three plant material samples were collected for each experimental object (biological repetitions) and two extracts were obtained for each biological sample and analyzed independently. Amounts of isoflavone aglycones and their glucosides were expressed in relation to the internal standard peak area and the sample mass.

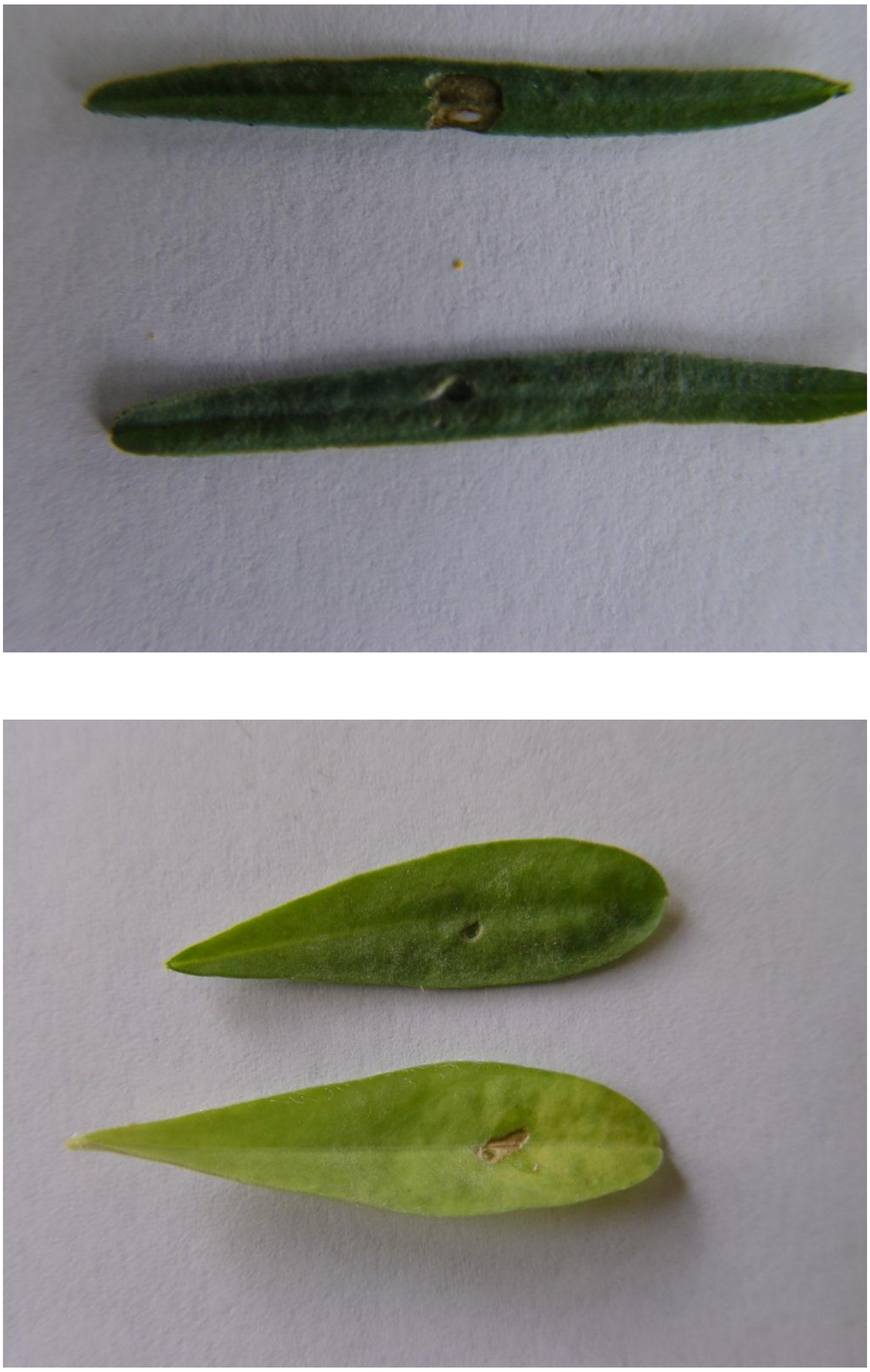

Figure 1S. Phytotoxicity of *Colletotrichum lupini* metabolites that were purified using reversed-phase C18 flash chromatography. The column eluate was spotted on needle-punctured leaves of *L. angustifolius* (top) and *L. albus* (bottom).

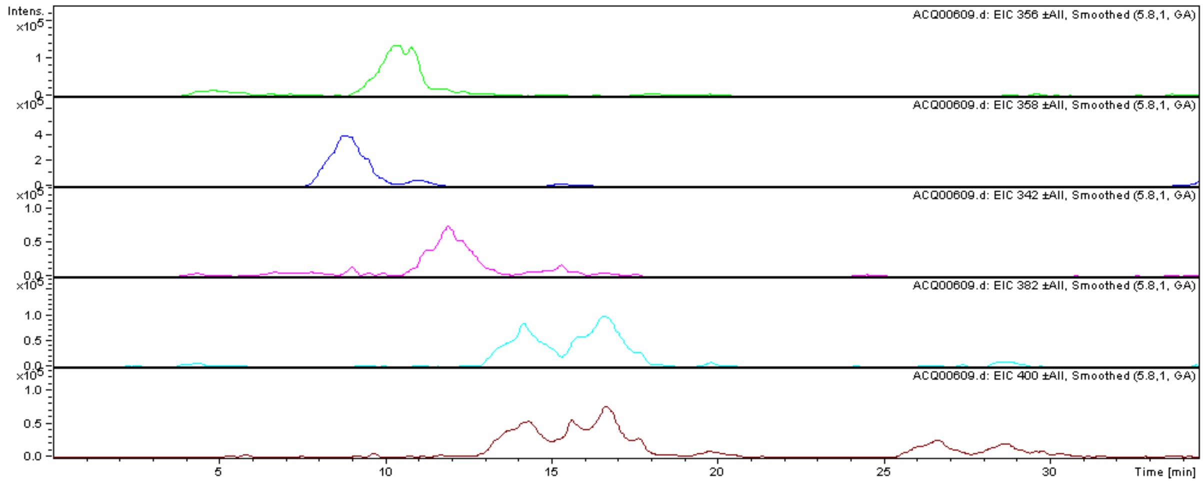

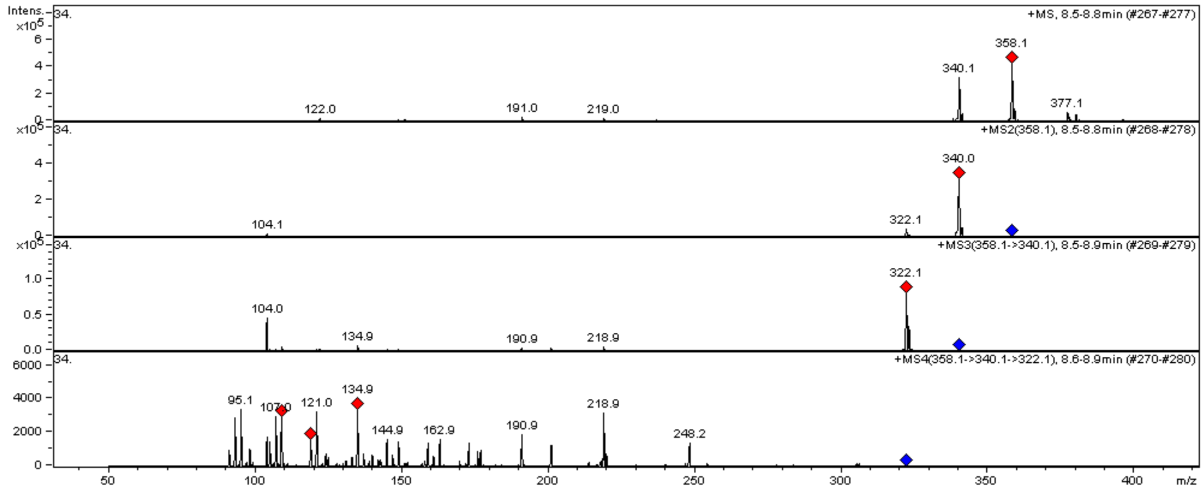

Figure 2S. HPLC-MS^n analysis of the phytotoxic fraction of the *Colletotrichum lupini* metabolites. Chromatograms were obtained in the positive ion mode and drawn for *m/z* A: 356; B: 358; C: 342; D: 382 and E: 400. The shown sequential MS spectra correspond to the $[M+H]^+$ ion at *m/z* 358.

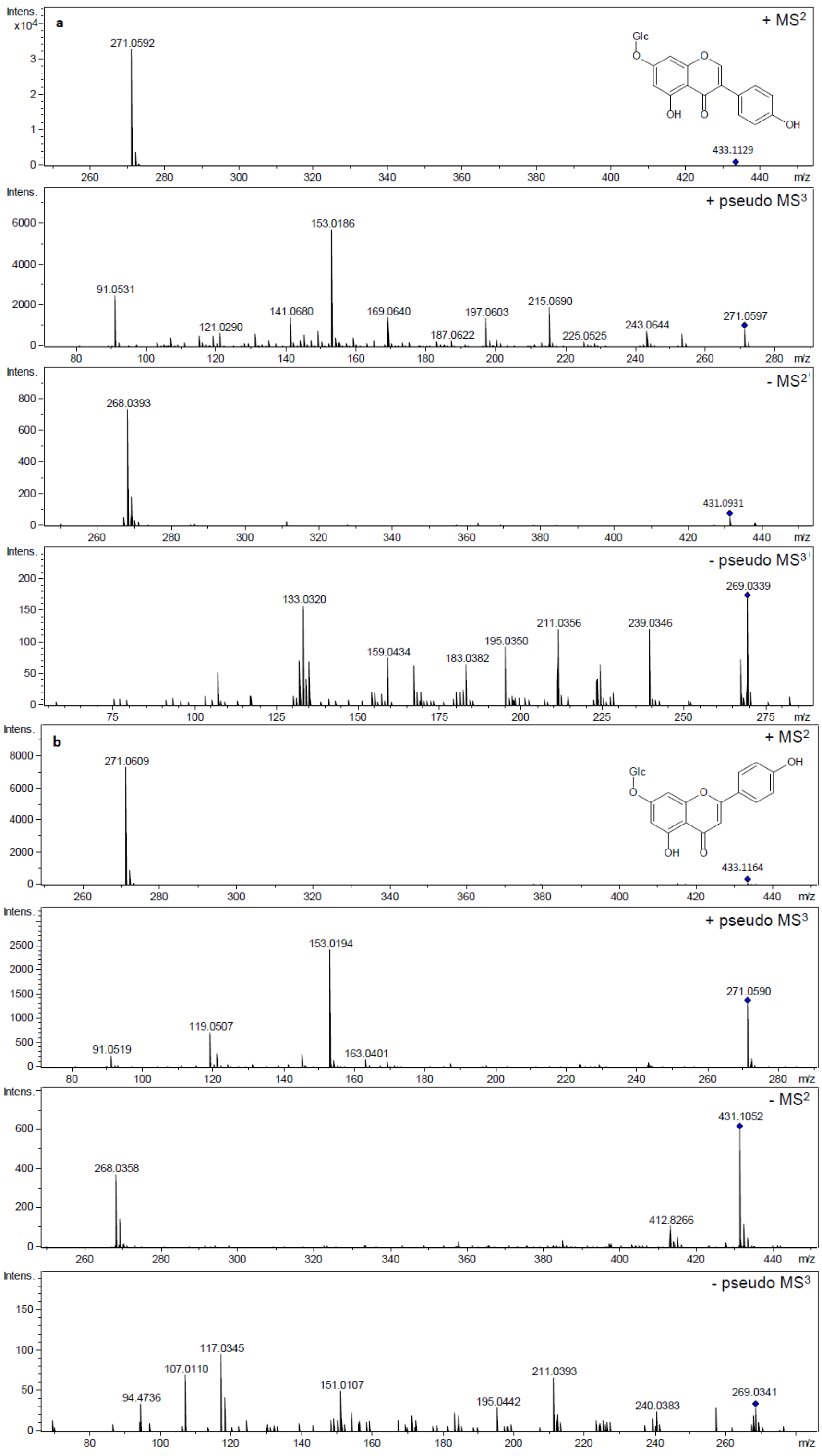

Figure 3S. Collision-induced mass spectra of flavonoid positional isomers genistein-7-*O*-glucoside [a]; and apigenin-7-*O*-glucoside [b] recorded in the MS^2 and pseudo-MS^3 modes for the positive $[M+H]^+$ (at *m/z* 433) and negative $[M-H]^-$ (at *m/z* 431) ions and the respective aglycone ions at *m/z* 271 and 269.

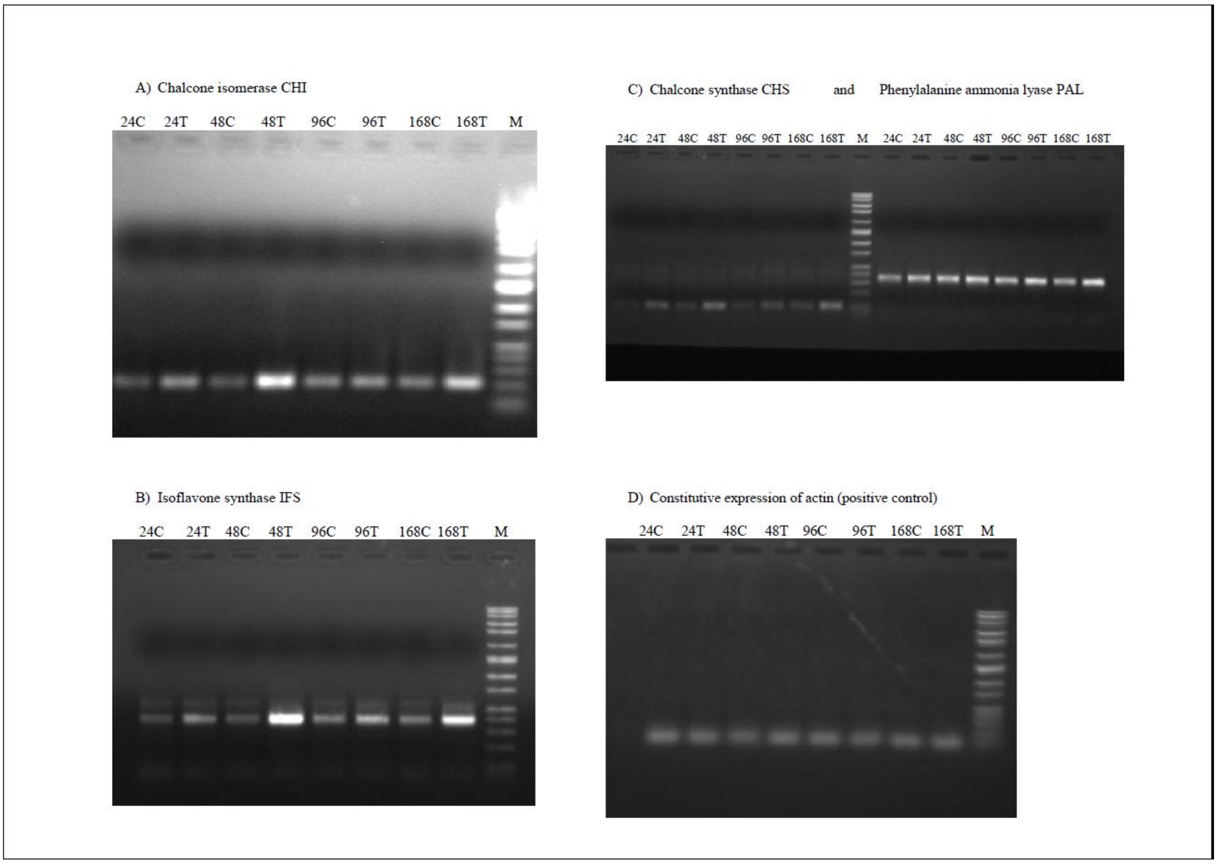

Figure 4S. Agarose gel electrophoresis of cDNAs corresponding to the transcripts of [A] chalcone isomerase (CHI); [B] isoflavone synthase (IFS) [C] chalcone synthase (CHS) and phenylalanine-ammonia lyase (PAL) genes and [D] the positive control – the actin gene. The reaction conditions for the reversed transcription and the PCR are described in the 'Materials and methods' section. cDNA samples were obtained in two experiments: spraying of lupin plants with the phytotoxin solution and collection of samples after 24 and 48 hours after the elicitation and spraying plants with fungal spores and collection of samples after 96 and 168 hours.

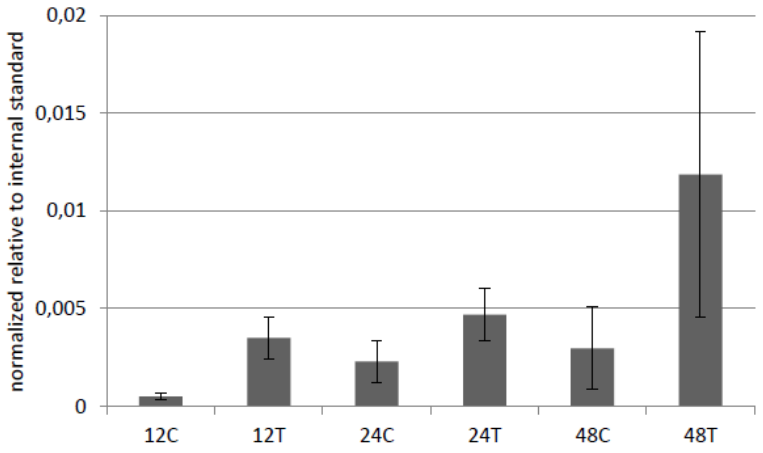

Figure 5S. Contents of wighteone detected in the samples obtained by washing of lupin leaves with CH_2Cl_2, obtained at three time points after elicitation (12 h, 24 h, and 48 h) of lupin plants (*L. angustifolius* cv. Sonet) with phytotoxin obtained from cultures of fungus *C. lupini* (T) and control plants (C).

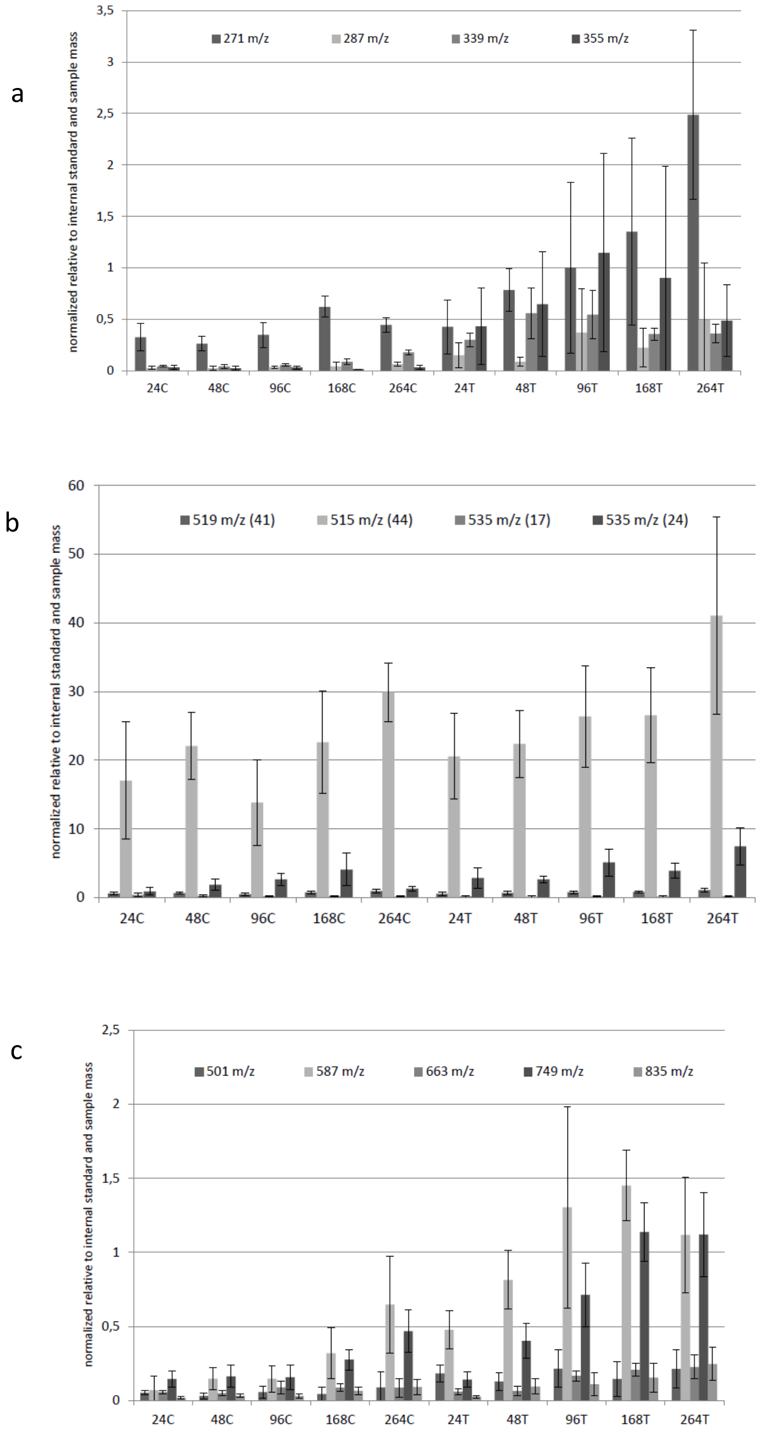

Figure 6S. Contents of chosen isoflavones and their glycoconjugates detected after infection with *C. lupini* spores of lupin plants (*L. angustifolius* cv. Sonet) 24 h, 48 h, 96 h, 168 h and 264 h after infection (T) and control plants (C). Free isoflavone aglycones: genistein – $[M+H]^+$ at *m/z* 271 (**65**), 2'-hydroxygenistein – $[M+H]^+$ at *m/z* 287 (**53**), wighteone – $[M+H]^+$ at *m/z* 339 (**72**), luteone – $[M+H]^+$ at *m/z* 355 (**71**) [a]; malonylated genistein-7-*O*-glucoside – $[M+H]^+$ at *m/z* 519 – two isomers (**41**, **44**), malonylated 2'-hydroxygenistein-7-*O*-glucoside – $[M+H]^+$ at *m/z* 535 – two isomers (**17**, **24**) [b]; wighteone glucoside – $[M+H]^+$ at *m/z* 501 (**68**), malonylated wighteone glucoside – $[M+H]^+$ at *m/z* 587 (**69**), wighteone diglucoside – $[M+H]^+$ at *m/z* 663 (**55**), malonylated wighteone diglucoside – $[M+H]^+$ at *m/z* 749 (**61**) and dimalonylated wighteone diglucoside – $[M+H]^+$ at *m/z* 835) (**63**) [c].

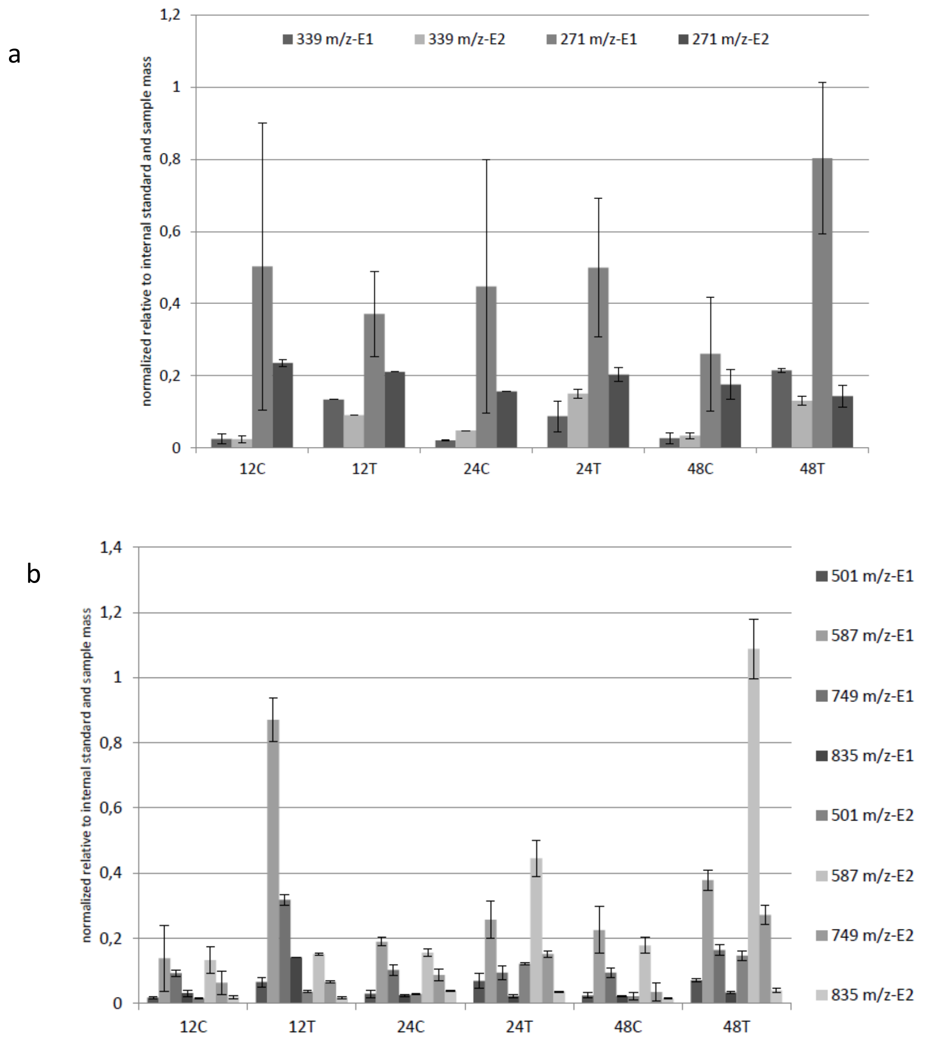

Figure 7S. Contents of wighteone ([M+H]$^+$ at *m/z* 339 - **72**) and genistein ([M+H]$^+$ at *m/z* 271 - **65**) [a] and wighteone glycoconjugates (wighteone glucoside [M+H]+ at *m/z* 501 (**68**), malonylated wighteone glycoside – [M+H]$^+$ at *m/z* 587 (**69**), malonylated wighteone diglycoside – [M+H]$^+$ at *m/z* 749 (**61**) and dimalonylated wighteone diglycoside – [M+H]$^+$ at *m/z* 835) (**63**) [b] in result of two types of elicitation of narrow leaf lupin plants with phytotoxins of *Colletotrichum lupini.* C: control plants, T: treated plants

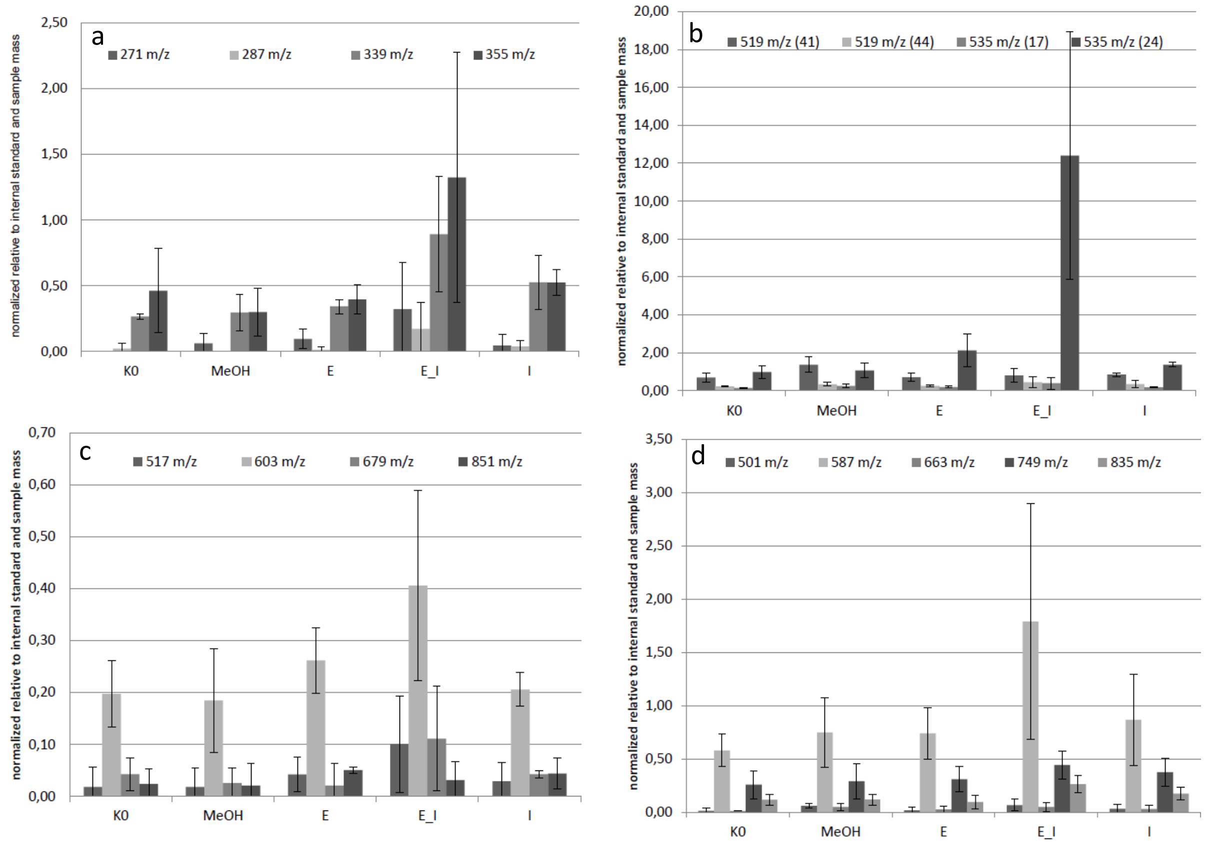

Figure 8S. Contents of chosen isoflavones and their glycoconjugates detected in 2 weeks old lupin seedlings (*L. angustifolius* cv. Sonet) after elicitation (**E**), infection with *C. lupini* spores (**I**) or elicitation followed with infection 48 hours after elicitation of lupin plants (**E + I**). Samples were also collected from control plants treated with water (K0) or methanol (MeOH). On the graphs are presented: relative amounts of free aglycones - genistein – [M+H]$^+$ at *m/z* 271 (**65**), 2’-hydroxygenistein – [M+H]$^+$ at *m/z* 287 (**53**), wighteone – [M+H]$^+$ at *m/z* 339 (**72**), luteone – [M+H]$^+$ at *m/z* 355 (**71**) [a]; isomers of malonylated glycosides of genistein and 2’-hydroxygenistein: (malonylated genistein-7-*O*-glycoside – [M+H]$^+$ at *m/z* 519 – two isomers (**41**, **44**), malonylated 2’-hydroxygenistein-7-*O*-glycoside – [M+H]$^+$ at *m/z* 535 – two isomers (**17**, **24**) [b]; luteone glucoside – [M+H]$^+$ at *m/z* 517 (**62**), malonylated luteone glucoside – [M+H]$^+$ at *m/z* 603 (**67**), luteone diglucoside – [M+H]$^+$ at *m/z* 679 (**45**) and dimalonylated luteone diglucoside – [M+H]$^+$ at *m/z* 851 (**60**) [c] and wighteone glyconjugates: wighteone glucoside – [M+H]$^+$ at m/z 501 (**68**), malonylated wighteone glycoside – [M+H]$^+$ at m/z 587 (**69**), and wighteone diglycoside– [M+H]$^+$ at *m/z* 663 (**55**), malonylated wighteone diglycoside – [M+H]$^+$ at *m/z* 749 (**61**) and dimalonylated wighteone diglycoside – [M+H]$^+$ at *m/z* 835) (**63**) [d].

Table 1S. Quinolizidine alkaloids identified on the leaf surface of narrow leaf lupin (*L. angustifolius*) plants.

M^+	Rt	Compound	PubChem ID
248	19.256	tetrahydrorhombifolin	3083799
234	19.623	angustifolin	21575185
248	19.890	α-isolupanin	119201
246	20.190	11,12-dehydrolupanin	618800
248	20.390	lupanin	91471
246	21.825	multiflorin	5319938
264	22.584	13-hydroxylupanin	73404
334	24.027	13-α-butyryl-oxylupanin	6428446
348	24.328	13-α-izovaleryl-oxylupanin	6428447
346	25.062	13-α-tigloyl-oxylupanin	6427214
346	25.429	13-α-angeloyl-oxylupanin	6427211
368	28.298	13-α-benzyl-oxylupanin	6430112
394	30.768	13-α-cis-cinnamoyl-oxylupanin	6427217
394	33.270	13-α-trans-cinnamoyl-oxylupanin	6427219
